# Mother-to-child transmission of human immunodeficiency virus in aten years period

**DOI:** 10.1186/1742-4755-8-35

**Published:** 2011-11-30

**Authors:** Adriane M Delicio, Helaine Milanez, Eliana Amaral, Sirlei S Morais, Giuliane J Lajos, João Luiz C Pinto e Silva, José Guilherme Cecatti

**Affiliations:** 1 Department of Obstetrics and Gynecology, School of Medicine, University of Campinas, Campinas, Brazil; 2 Centro de Referência DST/Aids de Campinas, São Paulo, Brazil

## Abstract

**Objectives:**

to evaluate mother-to-child transmission (MTCT) rates and related factors in HIV-infected pregnant women from a tertiary hospital between 2000 and 2009.

**Subjects and method:**

cohort of 452 HIV-infected pregnant women and their newborns. Data was collected from recorded files and undiagnosed children were enrolled for investigation. Statistical analysis: qui-square test, Fisher exact test, Student *t *test, Mann-Whitney test, ANOVA, risk ratio and confidence intervals.

**Results:**

MTCT occurred in 3.74%. The study population displayed a mean age of 27 years; 86.5% were found to have acquired HIV through sexual contact; 55% were aware of the diagnosis prior to the pregnancy; 62% were not using HAART. Mean CD4 cell-count was 474 cells/ml and 70.3% had undetectable viral loads in the third trimester. HAART included nevirapine in 35% of cases and protease inhibitors in 55%; Zidovudine monotherapy was used in 7.3%. Mean gestational age at delivery was 37.2 weeks and in 92% by caesarian section; 97.2% received intravenous zidovudine. Use of AZT to newborn occurred in 100% of them. Factors identified as associated to MTCT were: low CD4 cell counts, elevated viral loads, maternal AIDS, shorter periods receiving HAART, other conditions (anemia, IUGR (intra uterine growth restriction), oligohydramnium), coinfecctions (CMV and toxoplasmosis) and the occurrence of labor. Use of HAART for longer periods, caesarian and oral zidovudine for the newborns were associated with a decreased risk. Poor adhesion to treatment was present in 13 of the 15 cases of transmission; in 7, coinfecctions were diagnosed (CMV and toxoplasmosis).

**Conclusion:**

Use of HAART and caesarian delivery are protective factors for mother-to-child transmission of HIV. Maternal coinfecctions and other conditions were risk factors for MTCT.

## Introduction

Epidemiologic surveys show that HIV transmission patterns have changed, shifting the epidemic towards the poorer, those living in non-urban areas and women. Increasing heterosexual transmission is responsible for the ever growing rates of infected women. Considering that 85% of infected women are within their reproductive years, the risk of mother-to-child transmission (MTCT) is significant [1].

In Brazil, a sentinel study (Estudo Sentinela-Parturiente) has shown that the prevalence of HIV infection among pregnant women is 0.41% and that HIV testing for pre-natal care is performed in only 62.3% of patients [2]. Out of 63 countries analyzed by UNAIDS, Brazil provides antiretroviral therapy for 50 to 75% of infected pregnant women [1]. MTCT rates were evaluated in a large multicentric study that found 8.6% of newborn infection in 2000 and 7.1% in 2001 [3].

Several factors have been implicated in MTCT of HIV, including: advanced maternal AIDS-related illness, elevated viral loads (VL), breastfeeding, route of delivery, other infectious and obstetrics conditions and low CD4 cell counts during pregnancy [4-7]. Furthermore, viral subtype [8], viral concentration in maternal genital fluids [9,10] and genetic factors were also identified [11]. Short duration of antiretroviral therapy during the course of pregnancy, problems regarding adhesion to treatment [12] and coinfecctions such hepatitis C virus [13], genital herpes [14], cytomegalovirus [15], syphilis [16], toxoplasmosis [17,18] and recreational drug abuse [19] further increase the risk of MTCT.

The beneficial role of antiretroviral therapy in decreasing the risk of MTCT of HIV has been largely documented, since ACTG 076 protocols were published [20]. The use of highly active antiretroviral therapy (HAART) prior to conception and its earlier initiation in the course of pregnancy was also associated with lower rates of transmission [12].

CAISM/UNICAMP has been involved in the treatment and care of HIV-infected pregnant women since 1988. A historical cohort of HIV infected pregnant women has shown reduction of MTCT rates from 32.3% in 1990 to 2.9% in 2000. The greatest decline was observed after the introduction of the complete recommendations of ACTG 076 protocol. At that time, there were no reported cases of MTCT in pregnant women treated with HAART [21].

The objectives of this study were to evaluate mother-to-child transmission of HIV and related factors, to describe clinical and epidemiological profile of HIV infected pregnant women in a specialized prenatal care facility from 2000 to 2009 and to identify possible consequences of maternal infection to the newborn infant.

## Subjects and method

Observational and analytical study, with a historical cohort constituted by a population of 452 HIV-infected pregnant women in the period of 2000 to 2009 receiving care at the institution. The women were retrospectively selected from clinical records and from the epidemiologic surveillance system. A specific form was developed for data gathering and the EPINFO program was used for data analysis. The following variables were analyzed: epidemiologic characteristics of the women (age, formal education, parity, planned pregnancy), clinical characteristics (previous use of HAART, previous diagnosis of HIV, CD4 cell count, viral load, regimens of antiretroviral treatment, maternal coinfecctions and gestational problems) and characteristics of the newborn (birth weight, birth length, Capurro, Apgar scores, neonatal morbidity and use of oral zidovudine).

All newborns exposed to HIV were referred to the Pediatric Immunodeficiency Service at the same University. In the few cases, where a final diagnosis of HIV infection in the newborn was not possible due to loss of follow up, the main caretaker was contacted by phone or mail. Research protocol was previously approved by the Institution Review Board (Project number 459/2004).

Statistical analysis was conducted regarding demographic and epidemiologic characteristics of the women, prenatal care, use of antiretroviral therapy, delivery and newborn. Data was analyzed through proportions distributions and means, comparatively between groups of HIV-infected and non-infected children. The specific effect of several different interventions was analyzed through their individual risk ratios (RR). Possible associations between each variable were further tested with qui-square test or Fisher's exact test. The association between continuous variables was tested through student *t *test (parametric data), Mann-Whitney test (non-parametric data) and ANOVA. The 95% confidence interval and the level of significance of 0.05 were used. Data was analyzed with the statistical program SAS version 9.1.

The association between maternal and perinatal variables and MTCT was evaluated with qui-square test, Fisher's exact test and calculation of risk ratio and confidence intervals to 95%. Multivariate analysis was performed according to the COX model for proportional risks (adjusted risk ratio). However, due to problem during mathematical convergence related to the actual absolute number of positive outcomes (mother-to-child transmission events), results in this front could not be measured.

The study protocol was approved by the Internal Review Board (IRB) of the Center for Women's Integrated Healthcare (CAISM), Department of Obstetrics and Gynecology, University of Campinas (UNICAMP) and by the IRB of the School of Medical Sciences of the same institution (approval letter #459/2004).

## Results

**Figure 1:**
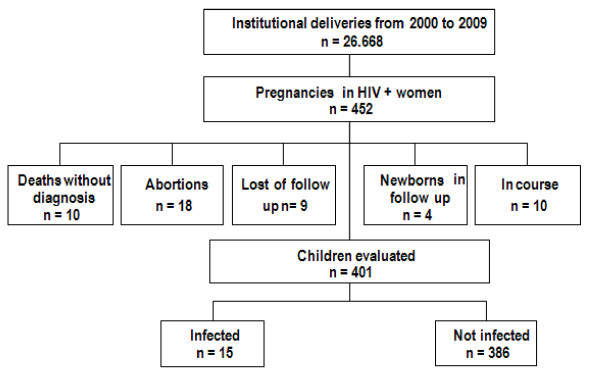
**Mother-to-child transmission in HIV pregnant women from 2000 to 2009**.

Between 2000 and 2009, 26.668 deliveries occurred in the institution and 452 were from HIV-infected pregnant women, corresponding to a prevalence of 1.69% of all deliveries. Figure 1 shows excluded cases in the final analysis, performed with 401 infants. Data referring to multiple gestations were not replicated. The analysis comprised 392 gestation events.

### Characteristics of pregnant women, prenatal care and delivery

The characteristics of pregnant women are presented in Table 1. The majority of them (243 in 389-62.5%) was not on antiretroviral therapy when they got pregnant. Among women already using antiretroviral drugs, the most common combination was two nucleoside reverse transcriptase inhibitors (NRTI) and one protease inhibitor (PI) or one non-nucleoside reverse transcriptase inhibitor (NNRTI). Ninety percent of women (295 in 328) reported good adhesion (95% or more of all HAART had been taken as prescribed, in two occasions) to treatment during pregnancy [22].

**Table 1:** Characteristics of HIV-infected pregnant women from 2000 to 2009

Characteristics		
	
	N	%
***Age***		
< 20	35	8.9
20 a 29	234	59.7
≥ 30	123	31.4
***Caucasian race***	234	59.7
***Number of pregnancies***		
1-2	177	45.2
≥ 3	215	54.9
***Fixed sexual partner***	263	76.9
***Planned pregnancy***	73	21.8
***Use of contraceptive method***	214	62.9
***Previous HIV-infected child***	30	10.6
***HIV diagnosis***		
Before pregnancy	215	55.1
During pregnancy	170	43.6
***Sexual contact transmission***	262	86.5
***CDC classification***		
1	102	26.2
2	161	41.4
3	72	18.5
Not classified	54	13.9
***First CD4 > 350 cel/ml (for NAIVE)***	137	39.1
***VL < 50(copies/ml) after HAART***	93	53.1
***Efavirenz exposition***	41	10.5
***Total***	392	100

(#) The numbers are different because of lack of informations from some patients

The mean number of prenatal visits was 7 (ranging from 1 to 18). Mean gestational age at the beginning of prenatal care was 18.1 weeks (ranging from 3 to 38). The majority of women (273 in 392-69.6%) presented at least one obstetric complication: preterm labor (14.5%), intrauterine growth restriction (6.1%) and chronic or gestational hypertension (5.1%) among others. Two hundred ninety nine in 392 (76.3%) had also at least one other infectious complication: bacterial vaginosis (32.9%), urinary tract infection (27%) and papilomavirus/intracervical neoplasia (22.7%) were the most prevalent. Resistance testing was performed only in 14 of 392 pregnant women (3.6%) by clinical indication (good adhesion and elevated viral load after two months of therapy). These patients were found to be carrying multiresistant HIV.

Prophylactic antiretroviral therapy (maternal CD4 > 350 for naive) was used in 39.1% (137 in 350). Only eleven in 392 women (2.8%) did not use antiretroviral therapy during pregnancy by late diagnosis; 41 in 384 (10.5%) were exposed to efavirenz. Zidovudine monotherapy was used by 28 in 384 women (7.3%) and in 21 patients, it was maintained until birth. ART combinations used are presented in Table 2. Mean CD4 cell-count was 474 cells/ml. Mean duration of antiretroviral therapy during pregnancy was 142.4 days. After HAART with PI, 66 pregnant women (71.7%) had undetectable viral loads. Three hundred and seventy in 380 cases (97.4%), intravenous zidovudine was used during labor and delivery.

**Table 2:** Association between HAART combinations during pregnancy and newborn infection

Newborn infection	With		Without		P*	RR	IC 95%
				
	N	%	n	%			
**Antiretroviral therapy**					**0.0478**		
AZT Monotherapy	3	14.3	18	85.7		1.00	
NRTI+NRTI	0	0.0	14	100		NC	
NRTI+NRTI+NVP	5	3.9	124	96.1		0.27	0.07-1.05
NRTI+NRTI+NFV	3	2.3	125	97.7		**0.16**	**0.04-0.63**
NRTI+NRTI+LPV/R	1	1.6	63	98.4		**0.11**	**0.01-0.99**
Other PI regimens	1	4.3	22	95.7		0.30	0.03-2.70

(#) Missed information for 13 cases in 392 gestation events

(##) NC: not calculated

(###) No antiretroviral: 11 cases; 2 cases with newborn infection

Mean gestational age at birth was 37.2 weeks. In 92% (357 in 388) of cases, delivery was conducted by caesarean section, mainly indicated by HIV infection.

### Characteristics of the newborns

Of 401 newborns, 208 (52.9%) were female. Mean birth weight was 2820 g and mean birth age by the method of Capurro was 37.6 weeks. Seventy nine children were premature (24%) and 87 (21.96%) had low birth weight. Neonatal use of oral zidovudine in the hospital was confirmed in 100% of cases.

Twenty one children presented with major congenital malformations among 48 in 393 (12.2%) presenting with any malformation: 9 cases of cardiac anomaly, 7 cases of genital tract malformations and 5 cases of urinary tract malformations. The remaining cases comprised minor malformation such as supernumerary finger, auricular appendage, among others.

Thirty four in 382 children (8.9%) presented at least one other illness during neonatal period (12 cases of pneumonia, 10 cases of hepatitis C, 6 cases of candidiasis and toxoplasmosis, 4 cases of cytomegalovirus infection, hepatitis B and urinary tract infection, 3 cases of meningitis and sepsis, 2 cases of syphilis and one case of rubella, varicela and tuberculosis).

### Factors associated with mother-to-child transmission of HIV

MTCT of HIV occurred in this cohort in 3.74% of cases. Risk factors identified for transmission were: low CD4 cell count, maternal AIDS-related illness, reduced time on HAART, obstetric and infectious concurring illnesses, presence of labor, neonatal coinfecctions, low birth weight, newborns small for gestational age, fail to complete postnatal prophylactic use of oral AZT and maternal and newborn anemia.

Any concurring AIDS-related illness during prenatal care enhanced the risk of transmission in around 5 times; maternal anemia (hemoglobin level under 11 g/dl) increased the risk of transmission in 4 times and also in cases of intrauterine growth restriction. Ovular infection elevated the risk in more than 21 times; neurotoxoplasmosis by itself increased it in more than 7 times (Table 3).

**Table 3:** Maternal factors associated and mother-to-child transmission of HIV

VARIABLES	MTCT With		MTCT Without		P*	RR	IC 95%
				
	N	%	n	%			
**AIDS-related illness**					0.0748		
Yes	2	14.3	12	85.7		**4.87**	**1.19-19.93**
No	11	2.9	364	97.1		1.00	
**First CD4 (cel/ml)**					**0.0029**		
≥ 350	1	0.5	212	95.5		1.00	
< 350	8	5.8	129	94.2		**12.44**	**1.57-98.35**
**Time using HAART**					0.0632		
15 days or longer	11	3.1	342	96.9		1.00	
Less than 15 days	2	16.7	10	83.3		**5.35**	**1.33-21.53**
**Ovular infection**					**0.0035**		
Yes	2	66.7	1	33.3		**21.61**	**8.15-57.29**
No	12	3.1	377	96.9		1.00	
**Neurotoxoplasmosis**					**0.0127**		
Yes	1	25	3	75		**7.46**	**1.26-44.23**
No	13	3.4	375	96.6		1.00	
**Route of delivery**					**0.0190**		
Vaginal	4	12.9	27	87.1		1.00	
Caesarean	10	2.8	347	97.2		**0.22**	**0.07-0.65**
**Labor**					**0.0088**		
Yes	6	9.8	55	90.2		**4.54**	**1.58-13.04**
No	7	2.2	316	97.8		1.00	
**Time after ruptured membranes**					0.5936		
Less than 4 hours	2	11.1	16	88.9		1.00	
> 4 hours	3	8.1	34	91.9		0.73	0.13-3.99
No	10		328				
**IV AZT**					0.3126		
Yes	12	3.5	362	96.5		0.35	0.05-2.40
No	1	10.0	9	90		1.00	
**Anemia (Hb level < 11 g/dl)**					**0.0254**		
Yes	4	11.8	30	88.2		**4.21**	**1.40-12.72**
No	10	2.8	348	97.2		1.00	

(#) The numbers are different because of lack of informations from some patients

First CD4 cell count during prenatal care was different among cases associated with transmission of HIV (219.1 cells/ml) and those not associated with it (480.9 cells/ml) (p = 0.002). Values lower than 350 cells/ml increased the risk of MTCT in more than 12 times (Table 3).

During gestation, use of HAART constituted protective factor against MTCT of HIV, with difference attributed to the mean duration of use in days among cases of transmission (93.2 days) and those without transmission (144.2 days) (p = 0.0121). HAART use for less than 15 days prior to delivery determined and increased risk of transmission of more than 5 times (Table 3). The use of antiretroviral drugs during pregnancy was not associated with low birth weight (p = 0.0808) or premature birth (p = 0.43).

Caesarean operation has shown protection in the order of 80% against MTCT of HIV. In the other, delivery occurring after the onset of labor determined an increased risk of almost 5 times for transmission of HIV (Table 3).

Children who were considered to be small for gestational age had an increased risk of almost 5 times for HIV infection. Mean values of birth weight was different in cases of transmission of HIV (2416 grams) and those without (2836 grams) (p = 0.0392). Low birth weight was associated with an increase of 3 times or more in the risk of transmission. Children who did not receive oral zidovudine in the neonatal period had an increased risk of acquiring HIV of more than 35 times. Neonatal infections were significantly associated with mother-to-child transmission of HIV. Toxoplasmosis increased the risk in almost 24 times and cytomegalovirus infection in more than 15 times (Table 4). Table 5 shows characteristics of HIV infected newborn and table 6 shows the mother-to-child-transmission rate year by year. The tendency is to a lower rate in the last years.

**Table 4:** Neonatal factors associated with mother-to-child transmission of HIV

VARIABLES	With		Without		P*	RR	IC 95%
				
	N	%	n	%			
**Birth weight**					**0.0270**		
≥ 2500 g	8	2.8	301	97.2		1.00	
< 2500 g	7	8.0	80	92		**3.11**	**1.16-8.33**
**Birth Weight in comparison to gestational age (GA)**					**0.0110**		
Adequate forGA	10	2.9	332	97.1		1.00	
Small for GA	5	13.2	33	86.8		**4.50**	**1.62-12.48**
**Oral AZT**					**0.0010**		
Yes	11	2.8	381	97.2		1.00	
No	2	100	0	0		**35.64**	**19.90-63.81**
**Neonatal pathology**					**0.0047**		
Yes	5	14.7	29	85.3		**5.67**	**2.01-15.96**
No	9	2.6	338	97.4		1.00	
**Congenital Toxoplasmosis**					**< 0.0001**		
Yes	4	66.7	2	33.3		**23.94**	**10.63-53.93**
No	11	2.8	384	97.2		1.00	
**Pneumonia**					**0.0076**		
Yes	3	25.0	9	75		**8.10**	**2.63-25.02**
No	12	3.1	377	97.9		1.00	
**Congenital CMV**					**0.0075**		
Yes	2	50.0	2	50		**15.27**	**5.00-46.63**
No	13	3.3	384	96.7		1.00	
**Hepatitis B**					1.0000		
Yes	0	0.0	4	100		NC	
No	15	3.8	382	96.2			
**Syphilis**					1.0000	NC	
Yes	0	0.0	2	100			
No	15	3.8	384	96.2			
**Anemia (hb < 13,5 g/dl)**					**0.0051**		
Yes	8	8.7	84	91.3		**5.83**	**1.58-21.46**
No	3	1.5	198	98.5		1.00	
**IUGR (intra uterine growth restriction)**					**0.0470**		
Yes	3	12.5	21	87.5		**4.18**	**1.25-14.00**
No	11	3.0	357	97		1.00	
**Hepatitis C**					1.0000		
Yes	0	0.0	10	100		NC	
No	15	3.8	376	96.2			

(#) The numbers are different because of lack of informations from some patients

(##) NC: not calculated

**Table 5:** Charateristics of HIV infected newborns.

Case	Year	PN visits	Initial CD4	Final CD4	Final VL	ARV	Time ARV	Adhesion	IV AZT	AZT newborn	Ruptured membran	Route of delivery	Labor	Capurro	Birth Weight	Maternal Pathology	Neonatal Pathology	Death
**01**	00	10	< 100	-	-	AZT	112	No	Yes	No	0	Ces	No	41	3370	No	PCP	Yes
**02**	00	-	-	-	-	AZT	49	No	Yes	Yes	2 h	Vag	Yes	36	2530	No	Toxo	No
**03**	00	2	-	-	-	AZT	40	No	Yes	Yes	0	Ces	Yes	40	3110	No	Rubella	No
**04**	01	8	-	-	-	AZT	21	No	Yes	Yes	0	Ces	No	38	3230	No	No	No
**05**	01	3	289	-	-	Haart	14	No	Yes	No	0	Ces	No	34	2635	No	No	No
**06**	01	9	11	05	47000	Haart	260	No	Yes	Yes	1 h	Vag	Yes	37	1985	Candid, PCP	Toxo	Yes
**07**	02	6	315	-	420	AZT	10	No	Yes	Yes	**240 h**	Vag	Yes	33	1320	Stevens-Johnson, Hep C	No	No
**08**	02	3	110	-	17000	Haart	255	No	Yes	Yes	0	Ces	No	40	3000	Neurotox	Toxo	No
**09**	03	5	344	-	< 50	Haart	140	**Yes**	Yes	Yes	0	Ces	Yes	40	3695	No	No	Yes
**10**	04	6	424	630	1410	Haart	84	No	Yes	Yes	0	Ces	No	35	1340	No	Oral cand.	No
**11**	04	5	233	-	-	AZT	135	No	Yes	No	0	Ces	No	33	1705	No	CMV	No
**12**	05	0	-	-	-	**No**	0	No	**No**	No	63 h	Vag	Yes	32	1425	No	No	No
**13**	06	2	160	-	-	Haart	21	No	Yes	Yes	0	Ces	Yes	39	2790	No	No	No
**14**	06	1	135	120	38380	Haart	-	No	Yes	Yes	7 h	Ces	Yes	37	2015	No	First gemelar	No
**15**	08	3	86	146	63	Haart	70	**Yes**	Yes	Yes	0	Ces	Yes	37	2100	Oral candid	Neurotoxo, CMV, TB	No

**Table 6:** Mother-to-child transmission of HIV by years of birth

YEAR	With		Without		total
		
	N	%	n	%	
**2000**	3	13.6	18	86.4	22
**2001**	3	6.1	46	93.9	49
**2002**	2	3	65	97	67
**2003**	1	2.5	39	97.5	40
**2004**	2	3	40	97	42
**2005**	1	3.1	31	96.9	32
**2006**	2	4.4	44	95.7	46
**2007**	0	0	37	100	37
**2008**	1	1.9	51	98.1	52
**2009**	0	0	12	100	12
Total (#)	15	3.7	384	96.3	399

(#) The numbers are different because of lack of information from some patients

## Discussion

The present study, analyzing 452 pregnancies from 2000 to 2009, found an HIV MTCT rate of 3.74%, which was above the expected for a reference maternity, in which nationally [23] and internationally accepted standard prophylactic measures are routinely employed in the care of HIV infected pregnant women. However, further studying of the 15 cases of transmission of HIV has shown particular results that explain such numbers. In spite of a multidisciplinary approach to prenatal care, with emphasis in medical (infectious disease specialist and obstetrician) and psychosocial care, the vast majority of mothers had substantial problems regarding adhesion to treatment and regular use of antiretroviral therapy prescribed during the gestation period. As a direct consequence of poor adhesion to treatment, some women reached delivery bearing high viral loads or even unknown values of viral load.

Our results shown several risk factors (low CD4 cell count, maternal AIDS-related illness, reduced time on HAART, obstetric and infectious concurring illnesses, presence of labor, neonatal coinfecctions, low birth weight, newborns small for gestational age, fail to complete postnatal prophylactic use of oral AZT and maternal and newborn anemia) associated with MTCT of HIV, confirming worldwide data on the subject [4-7,12,15,17,18].

Women with advanced degrees of imunossupression (CD4 < 350 or AIDS) had a significantly higher risk of transmitting HIV to their newborns, similarly to results found in literature [4-7,24].

HAART leads to undetectable viral load more rapidly in the course of treatment [12,25-27]. HAART in comparison to AZT monotherapy was strongly associated with decreased risk of MTCT. Duration of antiretroviral therapy shorter than 15 days was associated with and increased risk, as was demonstrated by international study [12]. It is established that after two weeks of antiretroviral therapy the decrease in maternal viral load would be sufficient to decrease the risk of neonatal infection. Our data found that PI containing regimens were an important factor to protect against MTCT.

Preterm and low birth weight were not observed as complications of HAART use during pregnancy, in agreement with findings of other studies [28-31], but as opposite to what was concluded by others [32-34]. Thus, our results confirm the safety of antiretroviral drugs during pregnancy.

Obstetric and infectious complications of pregnancy have also lead to an increase in the risk of newborn HIV infection, in particular: anemia, intrauterine growth restriction and ovular infection. These factors can be symptomatic of compromised placental barrier function, increasing its permeability to HIV [35] and corroborate the importance of referring prenatal care of HIV infected pregnant women to tertiary specialized healthcare centers.

Great controversy still exists regarding the route of delivery in HIV infected pregnant women. Micro transfusions occurring during uterine contractions enhance blood exchanges between mother and fetus and the presence of significant viral load becomes an important factor in newborn HIV infection. Another issue presents itself as plasma viral load does not adequately represent viral concentrations in vaginal fluids [9]. So, even patients with sustained undetectable plasma viral load could still carry substantial viral load in vaginal secretions. Our data pointed out that the presence of labor has significantly increased the risk of MTCT.

This study demonstrated also, in concordance to current literature, a lower rate of MTCT following elective caesarean delivery, including women on potent antiretroviral therapy and with low peripartum plasma viral load [25,36]. In fact, this has been the standard of care in this healthcare facility since 2005, provided that the patient did not present in advanced labor (elective caesarean delivery). Previous studies discussed the higher post partum morbidity in HIV pregnant women submitted to a caesarean section. However, recent studies did not find this association, leading to a relative safety by performing a caesarean in these women [25].

Premature rupture of membranes followed by vaginal delivery was present in one case of newborn infection, confirming it as a collaborating factor for MTCT [24,35].

Low birth weight and small for gestational age also had increased risk for MTCT [37]. Among 15 infected newborns, in seven cases low birth weight was a contributing factor. Furthermore, several of these presented with serious infectious complications of the neonatal period. Maternal acute infections can compromise placental barrier, increasing the risk of MTCT. Several studies have demonstrated association, especially with maternal toxoplasmosis [17,18] and CMV [15], reinforcing the need of exhaustive screening of these infections during prenatal care. Co-infection with hepatitis C occurred in more than 10% of cases, which can also enhance transmission of HIV [13].

There was one case of multiple gestation in which only the first to be born was infected. Although there is no known association between MTCT and multiple pregnancies after the use of HAART, the first newborn is more frequently infected [38].

Postnatal prophylaxis with oral zidovudine has demonstrated to be a significant factor in protection against neonatal HIV infection since ACTG 076 protocol [20], and our findings were similar.

This study demonstrates that MTCT was strongly associated with maternal factors such as the stage of HIV infection, represented by CD4 counts and viral load, use of HAART and route of delivery, and also factors regarding the gestational period, especially the presence of maternal infectious and obstetric complications. Regarding the newborn, use of neonatal zidovudine still poses as an important factor in the protection against HIV transmission.

One limitation of this study was the impossibility of multivariate analysis due to a limited number of infected newborns, which led us to interpretations based solely on risk ratios.

The increasing number of women infected with HIV observed over the past few years alerts to the importance of approaching the issue of MTCT as a serious public health problem. As a direct consequence of this are the emotional losses to these families and also the considerable financial expenses to both the public and private health system for the care and management of an incurable infection.

Our data demonstrate a high rate of MTCT. However it is strongly associated with poor adhesion to treatment of both HIV and concurring illnesses, to the presence of AIDS and other serious diseases. If only the cases in which newborn infection was in fact preventable were to be analyzed separately, it could be concluded that in one single case such scenario was true, in which all standard prenatal measures were implemented successfully leading to undetectable viral load prior to delivery.

The increasing number and complexity of cases reduce the quality of psychosocial and clinical support which, in turn, can influence adhesion to therapy and eventually lead to poor outcomes to the newborn. Thus, it becomes paramount for the HIV-infected pregnant women to be referred to a tertiary care center offering a multiprofessional approach aiming towards good adhesion to treatment and, ultimately, reducing mother-to-child transmission of HIV.

## Competing interests

The authors declare that they have no competing interests.

## Authors' contributions

AMD and HM participated in all steps of the study, including research planning, data collection, analysis and writing the manuscripts. SSM performed the statistical analysis. JGC performed the final revision. All authors gave suggestions, read the manuscript carefully, fully agreed on its content and approved its final version.
